# Zn isotope fractionation in a pristine larch forest on permafrost-dominated soils in Central Siberia

**DOI:** 10.1186/s12932-015-0018-0

**Published:** 2015-04-16

**Authors:** Jerome Viers, Anatoly S Prokushkin, Oleg S Pokrovsky, Alexander V Kirdyanov, Cyril Zouiten, Jerome Chmeleff, Merlin Meheut, Francois Chabaux, Priscia Oliva, Bernard Dupré

**Affiliations:** GET/OMP, UMR 5563 CNRS, Université Paul Sabatier-CNRS-IRD, 14 avenue Edouard Belin, 31400 Toulouse, France; V N Sukachev Institute of Forestry SB RAS, Akademgorodok 50/28, Krasnoyarsk, 660036 Russia; BIO-GEO-CLIM Laboratory, Tomsk State University, Tomsk, Russia; Laboratory of Freshwater and Marine Ecosystems, Institute of Ecological Problems of the North, UB RAS, Arkhangelsk, Russia; LHYGES/EOST, Université de Strasbourg – CNRS, 1 rue Blessig, F-67084 Strasbourg Cedex, France

**Keywords:** Tree, Soil, Bog, Moss, Seasons, Larix, Transport, Translocation, Uptake

## Abstract

Stable Zn isotopes fractionation was studied in main biogeochemical compartments of a pristine larch forest of Central Siberia developed over continuous permafrost basalt rocks. Two north- and south-oriented watershed slopes having distinctly different vegetation biomass and active layer depth were used as natural proxy for predicting possible future climate changes occurring in this region. In addition, peat bog zone exhibiting totally different vegetation, hydrology and soil temperature regime has been studied.

The isotopic composition of soil profile from Central Siberia is rather constant with a δ^66^Zn value around 0.2‰ close to the value of various basalts. Zn isotopic composition in mosses (*Sphagnum fuscum and Pleurozium schreberi*) exhibits differences between surface layers presenting values from 0.14 to 0.2‰ and bottom layers presenting significantly higher values (0.5 – 0.7‰) than the underlain mineral surface. The humification of both dead moss and larch needles leads to retain the fraction where Zn bound most strongly thus releasing the lighter isotopes in solution and preserving the heavy isotopes in the humification products, in general accord with previous experimental and modeling works [GCA 75:7632–7643, 2011].

The larch *(Larix gmelinii*) from North and South-facing slopes is enriched in heavy isotopes compared to soil reservoir while larch from Sphagnum peatbog is enriched in light isotopes. This difference may result from stronger complexation of Zn by organic ligands and humification products in the peat bog compared to mineral surfaces in North- and South-facing slope.

During the course of the growing period, Zn followed the behavior of macronutrients with a decrease of concentration from June to September. During this period, an enrichment of larch needles by heavier Zn isotopes is observed in the various habitats. We suggest that the increase of the depth of rooting zone, and the decrease of DOC and Zn concentration in soil solution from the root uptake zone with progressively thawing soil could provoke heavy isotopes to become more available for the larch roots at the end of the vegetative season compared to the beginning of the season, because the decrease of DOC will facilitate the uptake of heavy isotope as it will be less retained in strong organic complexes.

## Introduction

In the context of climate warming boreal forested regions with continuous permafrost attract special attention because they are likely to encounter the most important changes. The latest technical study from the IPCC (www.ipcc.ch and references therein) on climate change and water resources reports abnormal permafrost thawing [1,2], a longer growing season [3-5], an increase of subsurface water drainage [6] and foresees significant changes in the distribution of plants and their productivity for arctic and sub-arctic regions [4]. All these changes are likely to modify the fluxes of chemical elements, including carbon and metal micronutrients between the main reservoirs (soil-rock system, plants, aquatic domains, and atmosphere) [7,8]. Siberian forests are especially important in this regard because they store approximately 140 gigatonnes of carbon in above-ground biomass and at least three times more as soil organic matter [9-11]. However, the limiting factors in the development of these forests remain poorly understood. In contrast to the large number of articles devoted to macronutrients limitation in coniferous forests over permafrost, studies of micronutrients, especially the divalent metals, are notably scarce. Among the different metals, zinc is especially interesting because *i*) it is essential for plant growth similar to manganese, molybdenum and nickel [12], and *ii)* after iron, Zn is the most abundant metal in living organisms [13]. In addition to the availability of nutrients in soils [14-18] internal mechanism**s** such as nutrients resorption or leaf-span longevity sustaining plant growth by increasing the mean residence time of nutrients within the plants are also important [19].

Until now, measuring Zn concentrations in the whole plant biomass, sap solution and individual organs remained the basic technique used by scientists for studying Zn biogeochemistry [13]. Over the past decade, with the appearance of a new generation of MC-ICP-MS, stable isotope methods have been widely employed to characterize Zn transport mechanisms and sources within continental or marine environments [20-41] comprising field studies, in vitro experiments on plants, experimental studies on Zn isotope fractionation during interaction with mineral and organic compounds and paleo proxies. The present work extends our knowledge of Zn fractionation in soil-plant system to a previously unknown boreal larch forest that has developed on permafrost soils. Our general objectives were to constrain the use of Zn as a proxy for other metals in biogeochemical processes in Siberian larch forest and to test the possibility that Zn is a limiting micronutrient using a stable isotope approach [42]. The specific questions to be addressed in this study were as follows: 1) To what degree are Zn isotopes fractionated between soil and tree in the permafrost environment? 2) Does this fractionation occur during soil – plant transfer, or within the plant itself? 3) How do the properties of contrasting habitats (in terms of nutrients availability, soil moisture and plant productivity) affect Zn isotopes fractionation? Finally, 4) Is the pool of Zn mobilized by the tree in these contrasted environments changing during the growing season (due to thawing)? We conducted our study within the Tura pilot-site (Central Siberia) to address these questions. This site is ideally suited for isotopic research in terrestrial biogeochemistry of permafrost forested environments under climate warming scenario [16,42-48]. As a first working hypothesis, and following results of previous studies, we assume that Zn uptake by plants growing in the permafrost environment is essentially controlled by Zn availability in the frozen soil. At the beginning of the active season, when the soil is frozen, Zn concentration and isotopic signature in the tree will be determined by available stocks of Zn within the plant. Upon progressive thawing of soils, deeper horizons may provide Zn to the roots. The second working hypothesis is that one can use the difference in Zn geochemistry between the slopes of southern and northern exposition, exhibiting dramatically different temperature and soil depth conditions, as a proxy for Zn isotope evolution in ecosystem of Central Siberia under on-going climate change.

## Material and methods

### Sampling site

**Figure 1:**
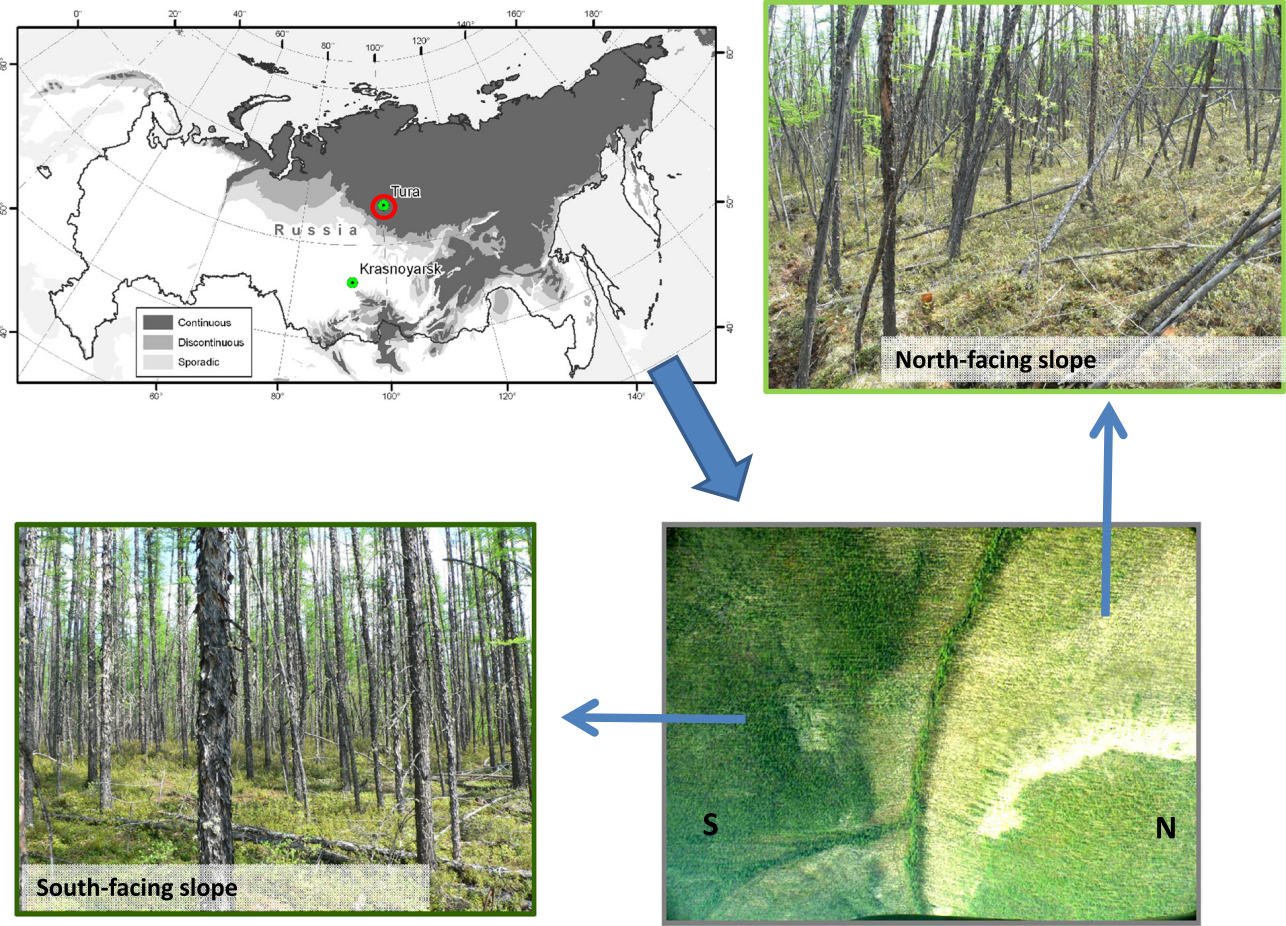
Map of Russia representing permafrost distribution (adapted from Brown et al., [81]) and map of the Kulingdakan catchment (64°17-19'N; 100°11-13'E) within the drainage basin of the Nizhniya Tunguska River (eastern tributary of the Yenissei River) on the basaltic plateau of Putorana (Central Siberia, Russia). Different environments (North-facing slope, South-facing slope, and sphagnum peat bog) of the Kulingdakan catchment are indicated. Detailed topographic map is available in Viers *et al*. [42].

Samples were collected in the Kulingdakan catchment (64°17-19'N, 100°11-13'E) of Central Siberia within the drainage basin of the Kochechum River, a northern tributary of the Nizhnyaya Tungunska River, which is the second largest tributary of the Yenissei River (Figure 1). This catchment area (~4100 ha) is located 5 km north of the town of Tura in Central Siberia, a region that extends to the east of the Yenissei River over more than 3,500,000 km^2^ at an elevation of between 130 and 1,200 m. The Kochechum River drains the basaltic rocks of the Putorana Plateau, which is a 248-Ma-old flood basalt complex cropping out over approximately 340,000 km^2^. Permafrost with a depth of between 200 and 400 m extends throughout the studied area (Brown et al., [81]). The soil is a fine-loamy mixed typic haplocryalf; further details regarding this region’s geology and soils are available in Pokrovsky *et al*. [49] and Bagard *et al*. [46,47].

The climate is cold and dry with a mean annual air temperature of −9.1°C (Tura Meteorological station, 1930–2013; Russian Research Institute of Hydrometeorological Information, http://www.meteo.ru). The air temperature varies from about −36°C in January to +16.5°C in July. The annual precipitation in this region is 369 mm, with 60-70% falling during the summer as rain and 30-40% as snow during the rest of the year. Snowmelt typically occurs during the second half of May. The amount of precipitation during the year 2006 was lower (272 mm) than the 1930–2013 average but the summer was particularly dry with only 25% of the annual precipitation. Soil thawing occurs from the end of May until September, during what is called the *"active period".* The vegetation of the study area is dominated by larch (*Larix gmelinii*), dwarf shrubs (*Ledum palustre L., Vaccinium vitis-idaea L.* and *Vaccinium uliginosum L.)* and mosses (*Pleurozium schreberi (Brid.) Mitt., Hylocomium splendens (Hedw.) B.S.G. and Aulocomnium palustre (Hedw.) Schwaeger*), along with patches of lichens [42,43]. For this study, we selected larch stands that developed after a strong ground fire in 1899 over three contrasting habitats, namely a South-facing slope, a North-facing slope, and a Sphagnum peat bog (Figure 1). These habitats differ in the following respects: 1) soil temperature and active layer thickness (ALT), 2) hydrological pathways, and 3) vegetation composition and biomass [43]. The main characteristics of the habitats are described below and listed in Table 1. South-facing slopes are characterized by a deep active layer and warm and well-drained soils with probable drought stress in mid-summer; the ALT depends on the year, but is generally approximately 120 cm for south-facing slopes. The total above-ground biomass (i.e., stem and crown) is ~57 t/ha, and the maximum rooting depth in the soil mineral layer is ~60 cm. North-facing slopes have cold soils with a maximum soil temperature at 5 cm < 5°C, a thin active layer with permanently moist soil from downslope suprapermafrost water flow. The maximum depth of the active layer in September is ~ 40 cm. The total above-ground biomass is ~28 t/ha and the maximum rooting depth in the soil mineral layer is ~10 cm. Finally, the peat bog had the shallowest ALT in mineral soil (~20 cm in September) with acidic and excessively moist conditions. The rooting zone is adjusted to the upper 15–40 cm of peat and does not reach the mineral layer. The total above-ground biomass is the lowest among the study habitats with ~7 t/ha. Nutrients in this area are mainly of atmospheric origin [50].

**Table 1:** **Plot, stand (2006) and soil characteristics of the Tura site (64°19' N, 100°15'E) in Central Siberia, modified after Prokushkin et al.** [44]

**Stand dominant**	**Tree stand**						**Soil**					
	**Age** ***(yrs)***	**Plot location**	**Stand density** ***(tree/ha)***	**Biomass** ***(t/ha)***			Active layer thickness** ***(cm)***				Organic layer*** ***(cm)***	**Max rooting depth in mineral layer** ***(cm)***
				**Stem**	**Needle**	Total*	**June**	**July**	**August**	**September**		
*L. gmelinii*	110	South-facing slope (SF)	2700	52.3	1.6	56.6	18 ± 8	86 ± 15	112 ± 26	123 ± 18	7 ± 2	60
*L. gmelinii*	110	North-facing slope (NF)	4400	26.9	0.4	28.2	1 ± 1	25 ± 12	36 ± 11	42 ± 10	19 ± 5	10
*L. gmelinii*	110	Sphagnum bog (B)	2483	5.6	0.3	7.0	0	1 ± 2	15 ± 8	20 ± 5	38 ± 8	0

*including branches; **at dates of needle sampling; ***including moss-lichen layer.

### Samples collection

Larch needle sampling was carried out in North and South-facing slopes and Sphagnum peat bogs (abbreviated as NF, SF, and PB, respectively) during the same growing season (June-September 2006). In each plot needle samples were collected from 3 larch trees of similar life status (diameter, height and crown development). From each tree we sampled the foliage of 3–5 mid-crown branches and composited them to one sample per tree. Sampling was performed on same trees four times during the growing season, starting with young needles that had attained their maximum length on June 7, continuing with mature needles at their maximum photosynthetic activity (July 18), followed by senescent needles (August 28) and was completed at the colored phase as needles started shedding (September 10). Shrinkage of needles before shedding was negligible. After collection, the plant material was cleaned on site with ultrapure water to remove possible aerosol dust particles attached to the surface and then stored in clean plastic bags [51]. The Zn concentrations were measured in the 3 tree samples of each microclimate environment and at each date. Each concentration value reported in the Tables 2 and 3 corresponds to the average and the standard deviation obtained from three independent analyses (Viers *et al.* [42], supplementary materials). The Zn isotopic compositions were measured on the composited sample obtained from the 3 samples of each microclimate environment and for the June, August and September periods. Based on our previous analysis of Zn and other metals in larch (Viers et al., [42]), these samplings correspond to 3 main periods of larch leaf formation and evolution allowing to assess the beginning of the nutrient mobilization, the maximal activity before the resorption, and the average isotopic composition of the larch litterfall.

**Table 2:** **Elemental concentrations (Zn, C, N) and Zn isotopic compositions for the different samples from the Kulingdakan catchment (Russia)**

**Samples**	**Environment type**	**[Zn]** ^**1**^ **μg/g**	**[C]** ^**2**^ **%**	**[N]** ^**2**^ **%**	**δ** ^**66**^ **Zn(‰)** ^**3**^	**δ** ^**66**^ **Zn(‰)** ^**4**^	**δ** ^**67**^ **Zn(‰)** ^**3**^	**δ** ^**68**^ **Zn(‰)** ^**3**^
***Soils***								
North0-10	*North-facing slope*	***120***	***4.02***	***0.14***	**0.27** ***± 0.03***	**−0.02**	**0.44** ***± 0.04***	**0.55** ***± 0.06***
North20-40	*North-facing slope*	***121***	***1.14***	***0.04***	**0.18** ***± 0.06***	**−0.11**	**0.25** ***± 0.07***	**0.34** ***± 0.12***
South20-40	*South-facing slope*	***108***	***0.71***	***0.02***	**0.24** ***± 0.02***	**−0.05**	**0.37** ***± 0.03***	**0.47** ***± 0.04***
South60-100	*South-facing slope*	***110***	***0.13***	***0.01***	**0.20** ***± 0.05***	**−0.09**	**0.30** ***± 0.09***	**0.41** ***± 0.08***
Bog0-20	*Sphagnum peatbog*	***108***	***3.35***	***0.17***	**0.21** ***± 0.02***	**−0.08**	**0.32** ***± 0.06***	**0.41** ***± 0.04***
***Sphagnum fuscum mosses***								
T1 (0–5 cm)	*Sphagnum peatbog*	***19.8***	***46.2***	***0.50***	**0.20**	**−0.09**	**0.39**	**0.40**
T1 (10–20 cm)	*Sphagnum peatbog*	***13.9***	***46.9***	***0.53***	**0.10**	**−0.19**	**0.01**	**0.32**
T1 (20–27 cm)	*Sphagnum peatbog*	***15.1***	***45.6***	***0.52***	**0.67**	**0.38**	**1.21**	**1.32**
T1 (28–32 cm)	*Sphagnum peatbog*	***14.7***	***45.7***	***1.19***	**0.57**	**0.28**	**0.91**	**1.13**
T2 (0–5 cm)	*Sphagnum peatbog*	***14.8***	***48.2***	***0.61***	**0.14**	**−0.15**	**0.28**	**0.30**
T2 (5–25 cm)	*Sphagnum peatbog*	***17.2***	***47.0***	***0.73***	**0.21**	**−0.08**	**0.24**	**0.47**
T2 (25–30 cm)	*Sphagnum peatbog*	***13.9***	***44.9***	***0.74***	**0.65**	**0.36**	**1.01**	**1.33**
T2 (30–32 cm)	*Sphagnum peatbog*	***20.7***	***40.7***	***1.20***	**0.55**	**0.26**	**0.96**	**1.12**
***Mosses (Pleurozium schreberi), litter and organic layer***								
Live (green) mosses (0–3 cm)	*North-facing slope*	***19.7***	***48.0***	***0.66***	**0.12** ***± 0.06***	**−0.17**	**0.36** ***± 0.09***	**0.35** ***± 0.12***
Dead (brown) mosses (3–6 cm)	*North-facing slope*	***25.5***	***48.1***	***0.31***	**0.03**	**−0.26**	**0.25**	**0.18**
Litter (7–10 cm)	*North-facing slope*	***17.1***	***48.1***	***0.80***	**0.41** ***± 0.04***	**0.12**	**0.72** ***± 0.06***	**0.87** ***± 0.07***
Organic layer (11–13 cm)	*North-facing slope*	***39.2***	***47.3***	***0.90***	**0.37**	**0.08**	**0.69**	**0.86**

The uncertainty reported for the Zn isotopic composition is the standard deviation obtained from duplicate or triplicate measurements depending on the amount of sample available. When no uncertainty was reported, only one measurement was performed. For these samples, the internal (relative) uncertainty of measurements was close to 0.05 ‰ (see Viers et al., ref. [22]). Otherwise the reported uncertainty is 2 s.d for *n* = 3.

^1^: Zn concentrations were measured on one sample that is composited (Mosses (Pleurozium schreberi), litter and organic layer) or not (soils, sphagnum fuscum mosses). The analytical error is < 10%.

^2^: C and N concentrations are from Prokushkin (in prep.).

^3^: Relative to the JMC Lyon (3-749 L).

^4^: Relative to the IRMM-3702 (Moeller et al., ref. [56]).

**Table 3:** **Elemental and isotopical composition of larch organs**

**Samples**	**Environment and date**	**[Zn]** ^**1**^ **μg/g**	**[C]** ^**2**^ **%**	**[N]** ^**2**^ **%**	**δ** ^**66**^ **Zn(‰)** ^**3**^	**δ** ^**66**^ **Zn(‰)** ^**4**^	**δ** ^**67**^ **Zn(‰)** ^**3**^	**δ** ^**68**^ **Zn(‰)** ^**3**^
*Needles*	*NF, June 7*	***38.4 ± 1.9***	***48.2 ± 0.8***	***2.66 ± 0.22***	**0.06** ***± 0.05***	**−0.23**	**0.17** ***± 0.09***	**0.17** ***± 0.08***
*Needles*	*NF, July 18*	***21.1 ± 6.9***	***48.6 ± 0.3***	***1.13 ± 0.09***	*n/a*	*n/a*	*n/a*	*n/a*
*Needles*	*NF, August 28*	***16.1 ± 3.6***	***48.7 ± 0.2***	***1.01 ± 0.12***	**0.22** ***± 0.01***	**−0.07**	**0.39** ***± 0.01***	**0.48** ***± 0.09***
*Needles*	*NF, Sept 10*	***11.8 ± 2.0***	***48.7 ± 1.2***	***0.23 ± 0.01***	**0.27** ***± 0.08***	**−0.02**	**0.37** ***± 0.14***	**0.46** ***± 0.18***
*Needles*	*SF, June 7*	***36.7 ± 13.6***	***47.8 ± 0.4***	***3.06 ± 0.40***	**0.20** ***± 0.01***	**−0.09**	**0.37** ***± 0.04***	**0.44** ***± 0.04***
*Needles*	*SF, July 18*	***12.6 ± 5.4***	***48.6 ± 0.5***	***1.47 ± 0.10***	*n/a*	*n/a*	*n/a*	*n/a*
*Needles*	*SF, August 28*	***14.5 ± 4.8***	***49.1 ± 0.4***	***1.21 ± 0.17***	**0.37** ***± 0.03***	**0.08**	**0.59** ***± 0.06***	**0.75** ***± 0.07***
*Needles*	*SF, Sept 10*	***13.3 ± 3.7***	***48.7 ± 0.8***	***0.31 ± 0.11***	**0.28** ***± 0.06***	**0.00**	**0.43** ***± 0.10***	**0.56** ***± 0.12***
*Needles*	*PB, June 7*	***36.1 ± 5.7***	***48.4 ± 0.5***	***2.23 ± 0.44***	**−0.21** ***± 0.10***	**−0.5**	**−0.22** ***± 0.16***	**−0.35** ***± 0.20***
*Needles*	*PB, July 18*	***20.9 ± 2.4***	***48.2 ± 0.8***	***1.27 ± 0.10***	*n/a*	*n/a*	*n/a*	*n/a*
*Needles*	*PB, August 28*	***13.3 ± 1.3***	***49.8 ± 0.3***	***1.04 ± 0.05***	**−0.15** ***± 0.05***	**−0.44**	**−0.16** ***± 0.07***	**−0.24** ***± 0.09***
*Needles*	*PB, Sept 10*	***18.2 ± 7.0***	***48.8 ± 0.7***	***0.26 ± 0.02***	**0.13** ***± 0.09***	**−0.16**	**0.21** ***± 0.12***	**0.26** ***± 0.18***
*Sapwood*	*SF, Sept 10*	***7.0 ± 2.3***	***48.4 ± 1.9***	***0.08 ± 0.01***	**0.47**	**0.18**	**0.62**	**0.92**
*Wood (bulk)* ^*5*^	*SF, Sept 10*	***4.8 ± 3.2***	***47.1 ± 3.3***	***0.10 ± 0.05***	**0.36**	**0.07**	**0.44**	**0.72**
*Bark*	*SF, Sept 10*	***23.3***	***51.9***	***0.15***	**0.73**	**0.44**	**1.14**	**1.48**
*Heartwood*	*PB, Sept 10*	***7.7 ± 2.4***	***43.9 ± 4.4***	***0.05***	**−0.06**	**−0.35**	**−0.17**	**−0.22**
*Sapwood*	*PB, Sept 10*	***15.8 ± 16.6***	***44.3 ± 3.4***	***0.07 ± 0.02***	**−0.15**	**−0.44**	**−0.24**	**−0.36**
*Wood (bulk)* ^*5*^	*PB, Sept 10*	***10.0 ± 7.1***	***47.1 ± 3.3***	***0.09 ± 0.02***	**0.00**	**−0.29**	**−0.01**	**−0.07**
*Fine roots (<2 mm) n = 5*	*SF, Sept 10*	***15.2***	*n/a*		**0.48**	**0.19**	**0.50**	**0.85**
*Phloem*	*riparian zone, Sept 10*	***60.5***	*n/a*		**0.67**	**0.38**	**0.90**	**1.21**

NF, SF and PB stand for North-facing slope, South-facing slope, and Sphagnum peatbog, respectively. The ± uncertainty is 2 s.d, where *n* = 5 to 10 for concentrations and 2 to 3 for isotopes. *n/a* stands for “non analyzed”.

^1^: Zn concentrations were measured on various composite samples; raw data are available in Viers et al. [42].

The Zn concentration is the average and the error the standard deviation.

^2^: C and N concentrations are from Prokushkin (in prep.).

^3^: Relative to the JMC Lyon (3-0749 L).

^4^: Relative to the IRMM-3702 (Moeller et al., ref. [56])*.* When no deviation standard is reported it signifies that only one sample was measured.

^5^: Wood bulk indicates heartwood + sapwood (= integral wood).

In September, all 3 trees from each plot were harvested to determine their above ground biomass. Stem discs (3 cm thick) were taken from every tree at breast height (1.3 m) to analyze tree ages and then different portions (parts) of the disc (bark, sapwood, heartwood and integral wood samples) originating from the South-facing slope and Sphagnum peatbog environments were taken for Zn concentration and isotopes analysis. Again Zn concentrations were obtained on the analysis of three samples while Zn isotopic composition was obtained on the composited sample.

In addition, in the slopes we have collected 5 replicate columns (0–13 cm depth) of surficial organic material divided into moss, *Pleurozium schreberii*, (separately live and dead portions), litter and soil organic layer. Replicates were composited to one sample. The Zn concentrations and isotopic compositions were measured on the composited samples. Within the Sphagnum peatbog site we sampled 2 peat columns (0–32 cm depths), which were divided into live Sphagnum fuscum moss layer (0–5 cm) and three layers of peat at different stages of decomposition (between 5 and 32 cm, see Tables 2 and 3). The Zn concentrations and isotopic compositions were measured on these samples.

Soil samples were collected just beneath the sampled organic columns in the South-facing slope, North-facing slope and Sphagnum peat bog in mid-August of 2006, at the period of maximum active layer thickness. Mineral soil sampling was performed by 100 cm^3^ cylinder for the entire active layer to the permafrost (0 to 120 cm). Further in the text, soil samples are indicated by the locality identifier followed by the sampling depth in cm (e.g., North0-10). In this example, "North" is the aspect of the soil sampling site and 0–10 indicates that the sample was collected from the 0 to 10 cm soil layer. Soil samples were passed through a 2 mm sieve and air-dried on site. Back at the laboratory, samples were dried at 80°C for 24 hours and finely ground using an agate mixer mill (Retsch, Germany). Given the extremely high homogeneity of the soil profile and the similarity between the unaltered basalt and the deepest (C) soil horizon [42,45,47,49,52], the mineral soil horizon sampled below the rooting depth, 60–100 cm at the South-facing slope, can be considered as unaltered mineral substrate. The bog site has no clear plant-related mineral substrate as the peat is permanently frozen below the rooting depth.

Interstitial soil solutions were collected both from N- and S-facing slope at different depths using suction cups and plate lysimeters, respectively, as described elsewhere [45].

Finally, the water samples from the large river (Kochechum) that drains this region [46,49] was collected in August 2006, filtered on site using trace clean techniques (a pre-washed Nalgene disposable filter unit with 0.22 μm acetate cellulose filter) and acidified with doubly distilled HNO_3_. An untreated MilliQ water sample was filtered to produce field blanks for analysis.

### Analyses

#### Sample preparation and Zn concentration measurement

To measure the elemental concentrations, all samples were digested in Teflon vials within individual polycarbonate compartments (A100) containing Teflon hot plates in a clean room (ISO3). Between 100 and 200 mg of plant and soil was first reacted with hydrogen peroxide (H_2_O_2_) for 24 hours at ambient temperature and further digested in HNO_3_ + HF, for 36 hours at 80°C, then in HCl for 36 hours at 80°C, and finally treated with HCl-HNO_3_ for 36 hours at 80°C.

Zn concentrations were measured by ICP-MS (Agilent 7500ce). Indium and rhenium were used as internal standards to correct for instrumental drift and eventual matrix effects. The international geostandards of basaltic rock BE-N (from CRPG, France) and lichens BCR-CRM 482 (from BCR, Belgium) were used to check the accuracy of the analytical methodology. The relative standard deviation between the certified values and our measurements were expressed as ([Zn]_certified_ - [Zn]_measured_)/(([Zn]_certified_ + [Zn]_measured_)/2)*100, and they were lower than 10% (1 s.d., *n* = 10). The agreement between our Zn analyses in the SLRS-4 standard of natural water and the certified value was better than 15% (2 s.d., *n* = 20). For isotopic analysis of the river water sample, large volume of filtered solution (~700 mL) was evaporated to dryness in the clean room under the hood box (A100) and the residue was subjected to acid leaching as for larch needles.

#### Zn separation and isotopic measurements

Zn was separated from the matrix using the AG-MP1 anion exchange procedure following Maréchal *et al*. [53] inside a laminar hood box ISO3 placed in the clean room. Two successive separations were performed for each sample. An aliquot of sample containing 800 ng was loaded on the column. Column loading was limited to <15% of the total anion exchange capacity of the resin. After the first separation procedure Zn fraction was loaded on a new column to perform a second separation (purification). Column yields was checked for each sample by analyzing Zn concentrations by quadrupole ICP-MS. Yields were found to be 95 ± 11% for Zn. Two samples presenting bad yields (<70%) were discarded and not considered in this study. Zn was removed using 0.05 M HNO_3_, the matrix used during MC-ICP-MS measurements. Note that this matrix is different from that used by Maréchal *et al*. [53] Total Zn blank due to the whole procedure separation are <8 ng which is insignificant relative to the total amounts loaded in the columns.

Zn isotopic composition was measured on a Thermo Finnigan NEPTUNE MC-ICP-MS hosted in GET (Toulouse). The Neptune introduction system used consists of a tandem quartz glass spray chamber (cyclone + standard Scott double pass) coupled with a low flow PFA nebulizer (50 to μL/min). Ar gas flows of ~15 L/min, ~1.2 L/min, and ~0.6 L/min were used for coolant, nebulizer and auxiliary, respectively. For ICP operation, an RF generator power of 1300 W was used. The beams were collected on a multi collector module with eight movable and one fixed Faraday cup displaying a maximum relative mass range of 17%. This cup configuration (used for Zn isotopic measurements) allowed simultaneous collection of the four Zn masses (64, 66, 67, and 68) ^63^Cu and ^65^Cu for mass bias correction and mass 62 to check for eventual Ni interferences [22]. After a minimum of 3 hours of instrument warm up and setting, analytical sequences were run automatically using a CETAC ASX-100 autosampler.

Between each sample and standard the machine was rinsed with 0.05 N HNO_3_ from two different vials for 1 minute each. Blank measurements consist of 1 block of 10 cycles (8 s) and sample and standard measurements consist of 2 blocks of 20 cycles of 8 seconds each. For sample and standard measurements the internal precision was found to be in the range 5–10 ppm (2σ err.) for both ^65^Cu/^63^Cu and ^66^Zn/^64^Zn. Instrumental mass fractionation was corrected by using two different methods: bracketing and internal standard [53,54]. A Cu standard (NIST 976) was added to the purified Zn fractions and a Cu (NIST 976) + Zn (JMC 3-0749 L) standard mixture was run as a brack standard, thus allowing to perform different corrections. The ^62^Ni signal was measured to evaluate the isobaric interferences of ^64^Ni on the ^64^Zn and no significant interferences were found in the samples. During one analytical session we checked that Cu and Zn fractionation factors remained constant. The Zn isotopic results in this study are given in the recommended delta notation for the ^66^Zn/^64^Zn ratio: δ^66^Zn = [((^66^Zn/^64^Zn)_s_ / (^66^Zn/^64^Zn)_JMC_)-1]*1000 (in ‰) where s stands for sample and JMC represents the Zn isotopic standard solution (JMC 3–0749 L) [55]. Note that this solution is not a referenced material but an elemental standard solution used by several laboratories [53]. The δ^66^Zn has been also expressed relative to the new certified reference material (IRMM-3702) for Zn isotopes measurements (see Tables 2 and 3) [56].

#### Mass dependent isotopic fractionation, analytical control and error evaluation

**Figure 2:**
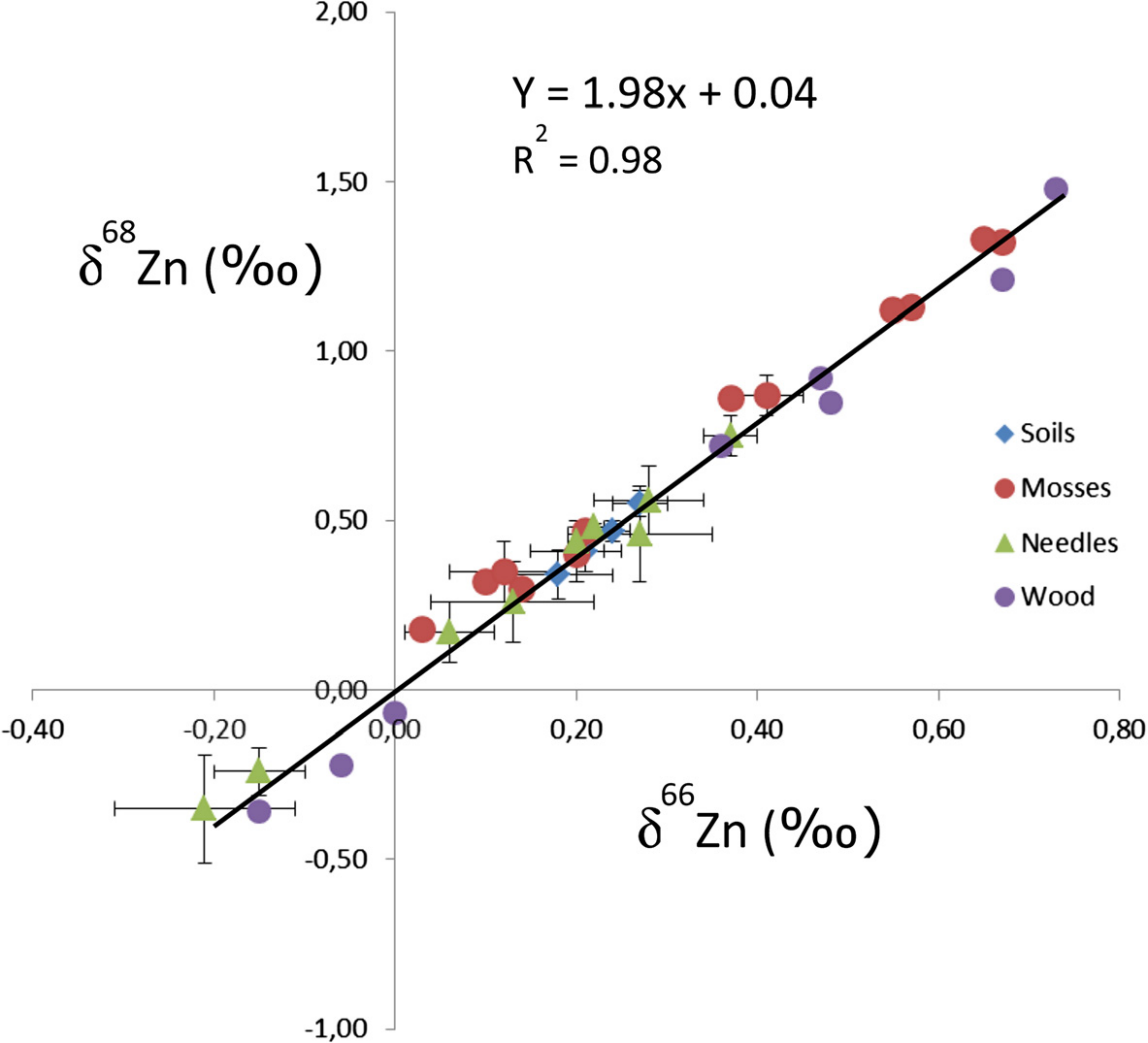
Isotope plot obtained during measurements of the samples using δ^6X^Zn= [((^6X^Zn/^64^Zn)_sample_ /(^6X^Zn/^64^Zn)_Lyon JMC 3-0749L_)- 1] * 1000 (in ‰).

The Figure 2 shows the δ^68^Zn (in ‰) as a function of the δ^66^Zn (in ‰) for the whole set of samples and demonstrate that the different Zn isotopes lie on the theoretical mass-dependent fractionation line. We observed that the slope is close to the theoretical slope of 2.000962. This figure reveals also that the ion exchange chromatography removed significantly all contaminants that could have induced isobaric interferences.

During the samples preparation, two reference materials (BCR-1 and CRM 482) were involved to check the external reproducibility. Our data (δ^66^Zn = 0.20 ± 0.07 for BCR-1 and δ^66^Zn = 0.10 ± 0.08) are in agreement with published values [21,22,57]. In the tables of results an error (standard deviation) is given for some of the samples when several replicates have been performed. Further details on the analytical precision of δ^66^Zn on standards during the analytical sessions, the analytical repeatability of a Zn monoelemental standard and procedural repeatability of the CRM standard as well as the long term measurement of Zn standards in our laboratory are presented elsewhere [22].

## Results

### Zn concentration and isotopic composition in soils and Zn concentration in soil solutions

The Zn concentrations and isotopic compositions of **5** selected soil samples are given in Table 2. We observed that both Zn concentrations (108 to 121 μg/g) and δ^66^Zn (0.18 to 0.27‰) are similar among different environments (south and north-facing slopes, and the bog) over the full depth of the soil column, except the upper mineral horizon of the north-facing slope exhibiting ca. < 0.1‰ heavier isotopic composition. The river water had a Zn isotopic composition (δ^66^Zn = 0.16 ± 0.05‰) similar to that of the measured soils.

**Figure 3:**
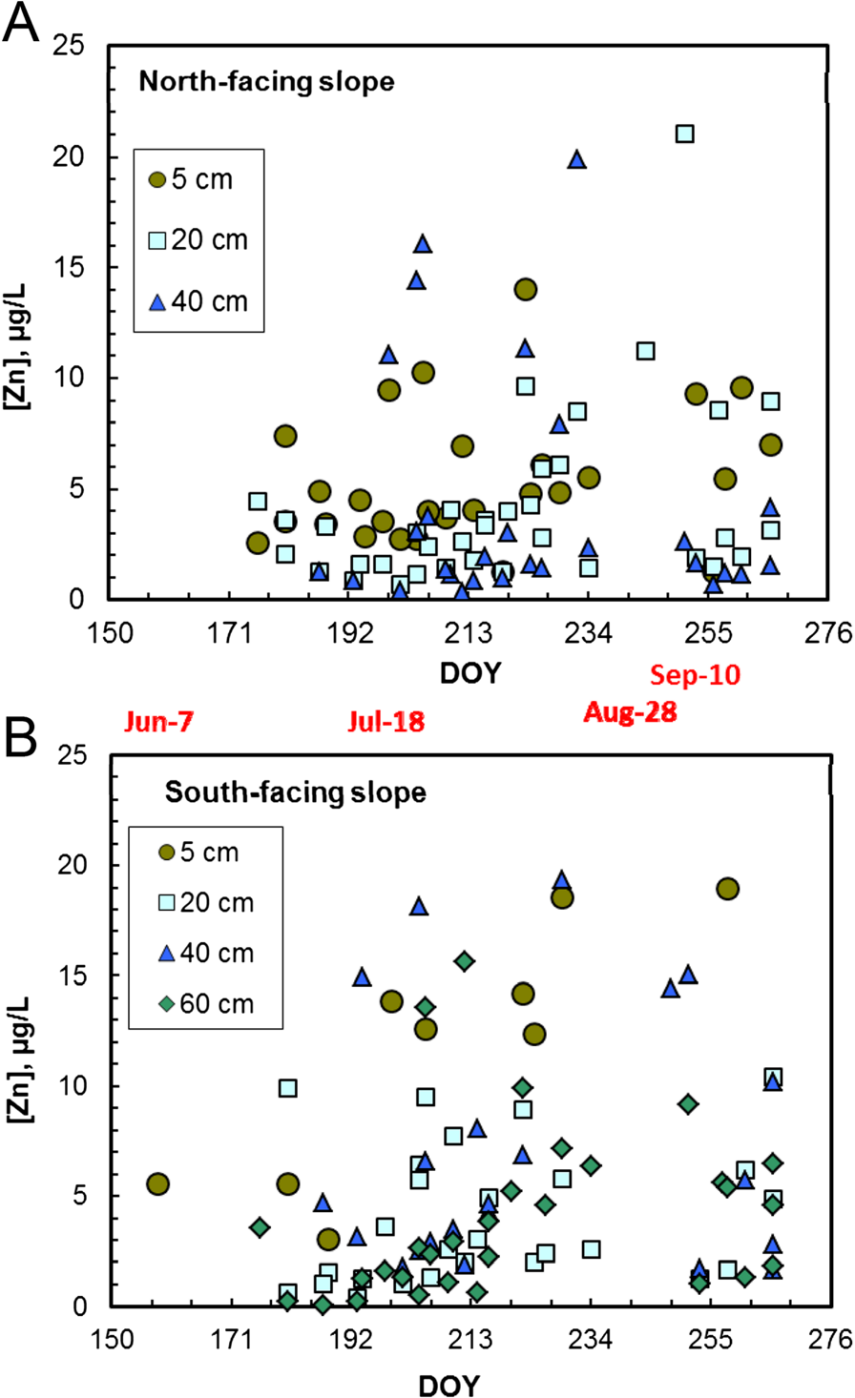
Zn concentration in interstitial soil solution at different depth as a function of the day of the year (DOY) measured in the N-facing **(A)** and S-facing **(B)** slopes.

**Figure 4:**
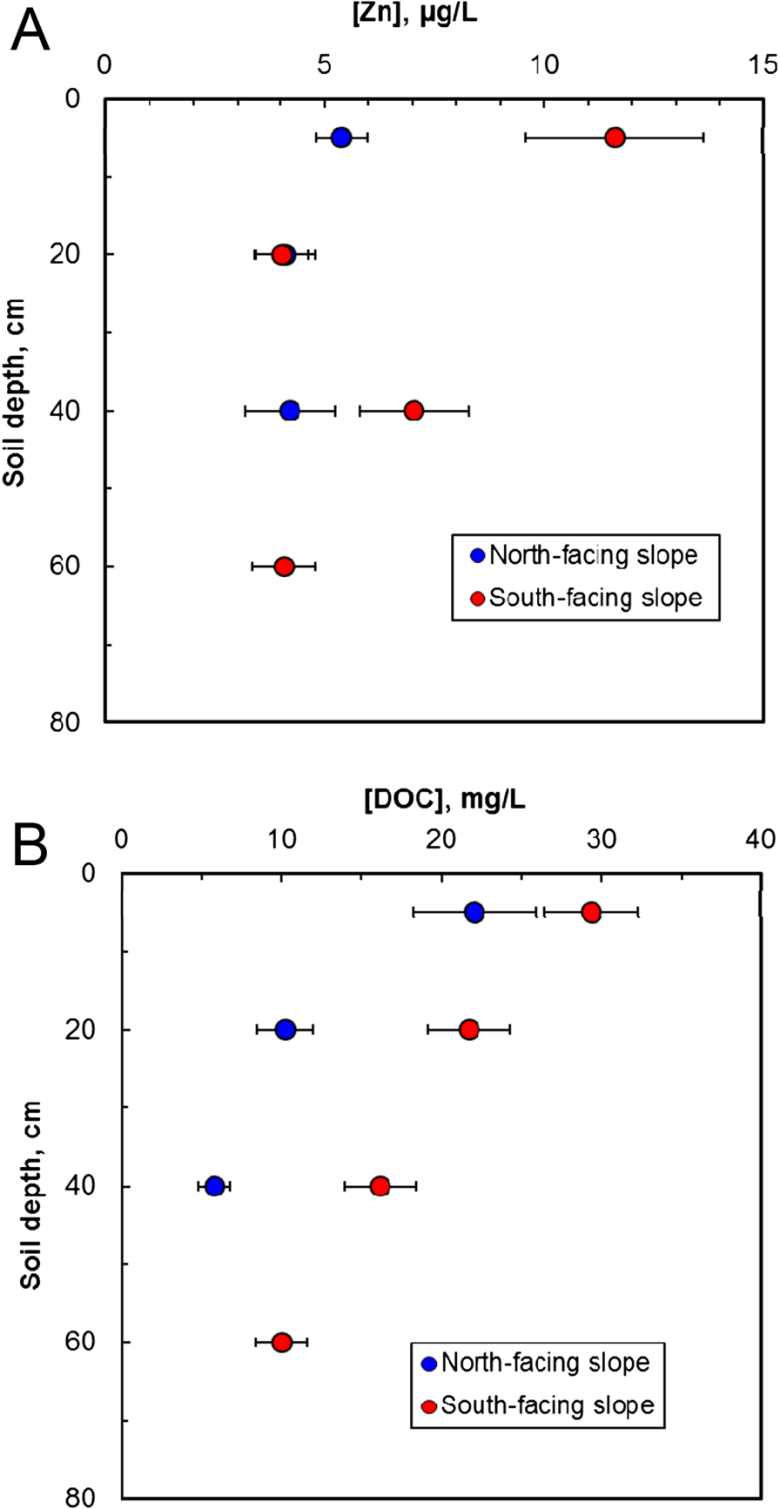
Average Zn **(A)** and DOC **(B)** concentration in soil solutions as a function of depth in the N-facing and S-facing slopes.

Zn concentration in soil solutions exhibited significant scattering over the active (unfrozen) period with lowest concentrations in the beginning of summer and the highest concentrations in the end of summer or in the autumn (Figure 3). This trend is clearly pronounced in the upper horizons on both slopes. On the annual scale, average Zn concentration in soil solution decreased from the surface to the bottom, following that of DOC (Figure 4A, B).

### Zn concentration and isotopic composition in mosses (*Sphagnum fuscum* and *Pleurozium schreberi*)

Samples from two microenvironments (trough and mound) of the *Sphagnum fuscum* peat bog (T1 and T2) were taken at different depths, ranging from the surface to 32 cm (Table 2). Zn concentrations ranged between 14 and 21 μg/g without any visible depth-related trend. In the T1 (trough) location, the peat Zn concentration was close to 20 μg/g in the most superficial sample and approximately 14 to 15 μg/g in the samples collected between 5 and 27 cm depth. In the T2 peat mound location, the Zn concentration was approximately 15 μg/g in the most superficial sample and between 14 and 21 μg/g in the other samples collected at greater depths. Again, we did not observe any relationship between Zn isotopic composition and Zn concentration in peat bog samples. However, δ^66^Zn increases steadily from 0.15-0.2‰ in the most superficial samples to around 0.6‰ at 32 cm depth.

The green (alive) and brown (dead) parts of *Pleurozium schreberi* moss exhibited a δ^66^Zn value close to 0‰. In comparison, litter and organic layer horizons developed beneath the moss exhibited distinctly heavier isotopic compositions from around 0.4‰, similar to the deeper layers of the sphagnum peat column.

### Zn concentration and isotopic composition in larch needles and wood 

**Figure 5:**
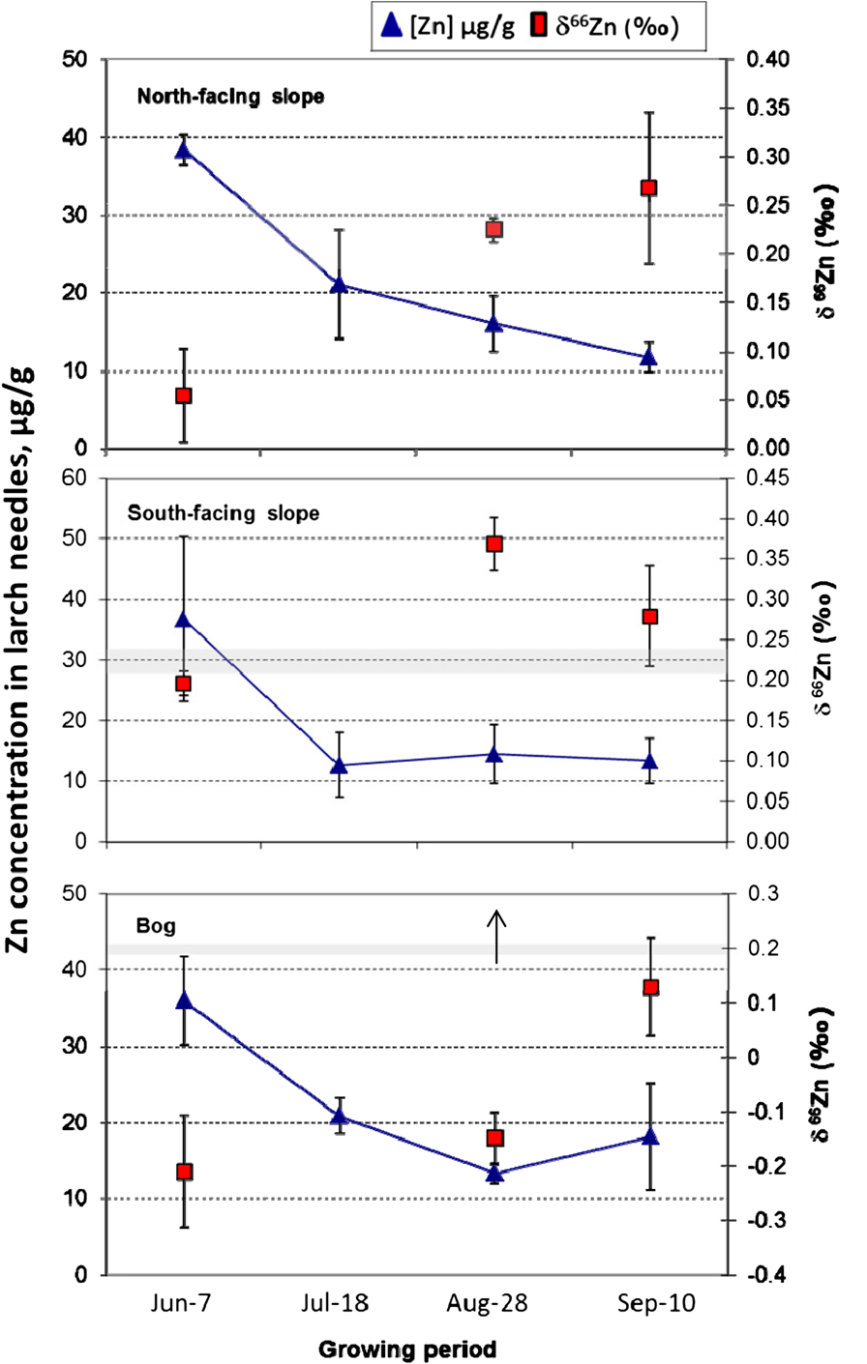
Concentration and isotopic composition of Zn in larix needles as a function of time during the growing period (June-September 2006). The shaded area represents the data for the nutritive reservoir of roots (mineral soil for both North- and South-facing slope and peatbog for bog area).

The Zn concentration and isotopic composition in the larch needles from different environments are listed in Table 3 and illustrated in Figure 5 as a function of the season (from June to September 2006). Due to the lack of sufficient plant material for the samples collected in July, we were unable to measure Zn isotopes for this month. However, the statistical treatment (unconstrained PCA) which was performed on the larch needles collected during 4 months in 3 locations in duplicates revealed that there are only two distinct periods in terms of metal micronutrient concentration: June and the rest of the summer (July, August, September) [42]. In the North-facing slope, South-facing slope and bog, we observed a Zn concentration change in larch needles as a function of time during the growing season. In the North-facing slope, the Zn concentration decreased from June (38.4 ± 1.9 μg/g) to September (11.8 ± 2.0 μg/g). The largest fraction of this decrease occurred between June and July. The Zn isotopic composition (δ^66^Zn in ‰) followed the opposite tendency with an increase from June (0.05 ± 0.05‰) to September (0.27 ± 0.08‰). In the South-facing slope, the Zn concentration decreased from June (36.7 ± 13.6 μg/g) to July (12.6 ± 5.4 μg/g) and then remained rather constant until September. Again, the δ^66^Zn (‰) increased from June (0.20 ± 0.02‰) to August (0.37 ± 0.03‰) and September (0.28 ± 0.06‰). In the bog area, the Zn concentration decreased from June (36.1 ± 5.7 μg/g) to September (18.2 ± 7.0 μg/g). As in the South- and North-facing slopes, the δ^66^Zn in peat bog larch needles slightly increased from June (−0.21 ± 0.10‰) to August (−0.15 ± 0.05‰); a much more significant increase occurred in September (+0.13 ± 0.09‰). Finally, the amplitude of the isotopic composition variation is 0.22‰, 0.17‰, and 0.34‰ for the North-facing slope, South-facing slope, and bog environments, respectively.

Comparing the isotopic signature of larch needles between the three different environments revealed a distinct difference between the bog area and valley slopes (Table 3 and Figure 5). Larch needles collected in August at the North and South-facing slopes exhibited δ^66^Zn values (0.22 to 0.37‰) close to those of the soils (0.18 to 0.27‰). In contrast, larch needles from the peat bog area exhibited much lower isotopic signatures (−0.21 to 0.13‰), which were especially pronounced in August.

**Figure 6:**
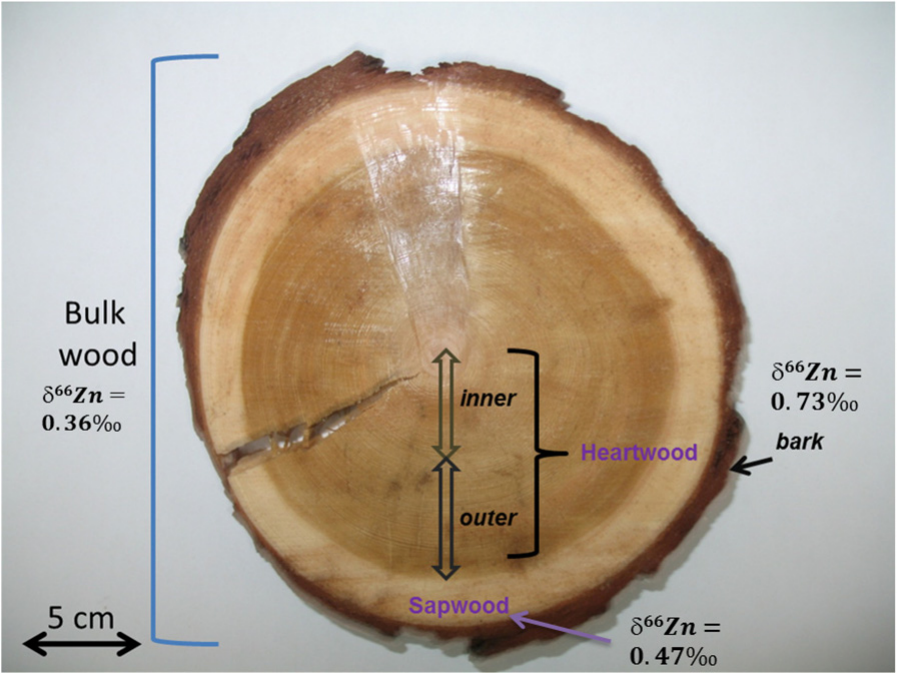
Different types of wood samples (bark, sapwood, heartwood and integral wood sample) taken from a stem disc at the end of the growing period (September 2006).

Zn isotopic measurements of the larch woody tissues (bark, sapwood, heartwood and the bulk sample, Figure 6) collected from the South-facing slope and in the sphagnum peat bog are listed in Table 3. It should be noted that the wood from these two different environments exhibited different isotopic compositions. Larch wood from the South-facing slope presented δ^66^Zn ranging from 0.36 to 0.73‰ and the wood collected in the bog area exhibited a much lighter Zn isotopic composition (from −0.15 to −0.00‰). The bulk wood from the bog (δ^66^Zn ~0‰) is isotopically lighter compared to the bulk wood from South-facing slope (~0.36‰); a similar trend was noted for the sapwood or heartwood (Table 3). Finally, the fine roots sample showed δ^66^Zn values close to 0.48‰ and the phloem isotopic composition (0.67‰) was close to that of the bark value (0.73‰).

## Discussion

### Zn isotopic composition in soils

The isotopic composition of soils from Central Siberia is notably constant with a δ^66^Zn value approximately 0.2‰. Although the isotopic composition of the basaltic rock constituting the geological substratum in Central Siberia is not available, the values of various basalts are highly homogeneous (δ^66^Zn = 0.20 ± 0.09‰ for BCR-1 standard [57]; δ^66^Zn = 0.24 ± 0.01‰ for 5 basaltic rock samples [58]; δ^66^Zn = 0.23‰ for BCR-2 standard [31]) and similar to those of the Tura bottom soil horizon (0.20 ± 0.05‰).

Results from the Tura site in Central Siberia indicate that there is no measurable Zn isotopes fractionation between parent rocks and soils assuming that *i*) the parent bedrock is similar to the C horizon of the soil, as it follows from other isotopical [45,47] and chemical [42] data, and *ii*) the Siberian basalts exhibit the same Zn isotopic signature as other basalts worldwide.

A homogeneous Zn isotopic composition over the full depth of the soil column corroborates previous studies on basalt chemical weathering in this region based on chemical and mineralogical analyses of rocks and soils and fluid solution chemistry [49,52]. These authors demonstrated that the weathering of basaltic rocks does not lead to chemical and mineralogical differentiation along the soil profiles under the current ecosystem state. As such, the fractionation of Zn isotopes in the soil profile is also minimal. The most likely mechanism for such a homogeneous composition is seasonal freezing front migration, downward in the beginning of the active season and both downward and upward in the autumn. This intensive mechanical mixing of the frozen/unfrozen soil horizon is further enhanced by sliding of the unfrozen soil layers along the ice boundary, especially visible at the N-facing slope. The observation that the whole soil profile is involved in seasonal cycles of freezing-thawing is also supported by high variability of Zn concentration in the interstitial soil solution in the course of the active season, sampled at different depths of the mineral layer, from 5 to 40 cm and from 5 to 60 cm on the NF and SF sites, respectively (Figure 3). Noteworthy a decrease of Zn concentration at the end of the active season, in September, observed on both environments. The averaged over all seasons Zn concentration in soil solution demonstrates the enrichment in surface horizons (5 cm) on the S-facing slope but virtually identical concentration over the full soil column in the N-facing slope, consistent with much higher water content of the NF habitats [43] and possible impact of the plant litter in productive SF habitats on Zn enrichment in the most surface soil horizon.

### Zn isotopic composition in mosses

Surface samples of live (green) mosses from *Sphagnum fuscum* peat bogs exhibited much lighter isotopic compositions compared to deep (brown) samples representing decomposed biomass. For the *Pleurozium schreberi* moss column, heavy isotope enrichment was also recorded in the litter and organic layer relative to the live and dead biomass of surface moss samples. Because there is no local contamination from an industrial complex, road or urban areas in the vicinity of our site, the δ^66^Zn values of the moss surface layers (from 0.14 to 0.20‰) may represent the background atmospheric deposition for this region [59]. Indeed, various studies have shown that mosses can be considered as a time-integrated measure of both wet and dry metal deposition from the atmosphere [60,61]. The δ^66^Zn values obtained from the bottom layers of the peat core (0.5 to 0.7‰) are significantly higher than the underlying mineral substrate. This heavy isotope enrichment may be related to the decay of organic matter induced by bacteria and fungi as may follow from Weiss *et al*. [62], although these authors did not evidenced this directly. A similar fractionation tendency towards heavy isotope enrichment in bottom horizons has been reported for carbon, nitrogen and sulfur in sphagnum peat bogs from a pristine area of the Czech Republic [63]. According to the latter authors, various other processes, such as nutrient recycling, ion exchange and absorption, may be involved in changing the initial isotope ratio.

Even if the main processes controlling isotope fractionation could be different between major nutrients and Zn, the retention of heavy isotopes during the humification process appears to be consistent with recent studies on Zn complexation, adsorption, and incorporation within biological and organic compartments [39,64-66]. Jouvin *et al*. [65] showed that the complexation of Zn by high molecular weight organic compounds (i.e., humic and fulvic acids) favors the heavy isotopes. Similarly, adsorption onto biological surfaces leads to cell enrichment with heavy isotopes as a consequence of the change in Zn coordination numbers and bond distances [64]. During humification process the labile products such as carbohydrates and amino acids are destroyed while aromatic structures appear [67,68]. Within that framework, and considering that chemical bonds Zn-O-C are stronger within the aromatic structures, in general accord with previous experimental and modeling works [1] we suggest that humification of both dead moss and larch needles leads to the enrichment of residual fraction by heavier isotope while releasing the lighter isotope in soil solution although direct field verification of this hypothesis is still lacking.

### Zn isotopic fractionation between soil and plants in different habitats

A larch root sample exhibited 0.2-0.4‰ heavier isotopic composition relative to the needles. Despite limited sampling, this result is in agreement with previous studies showing that roots are preferentially enriched in heavier isotopes relative to plant tissue following the conceptual model for Cu and Zn isotope fractionation during uptake at the root surface (Jouvin *et al*. [35] and references therein). According to these studies it is mostly agreed that *i)* within the root epidermal cell, Zn^2+^ ions and its organic and inorganic complexes can move via non-specific transport pathways favoring light isotopes; and *ii*) transport via specific Zinc/Iron regulated Proteins (ZIP) or phytosiderophores (PS) favors heavy isotopes [69]. A possible reason for the ^66^Zn enrichment in the roots is Zn-phosphate binding as supported by ab-initio calculations [70].

If we consider the root-shoot translocation, our results corroborate previous works showing heavy isotope depletion from root to shoot [33]. The translocation of metal by the xylem from roots to shoots occurs in the form of free Zn^2+^ and its complexes with organic acids (amino acids and peptides) when produced by the plant itself. The diffusion of these Zn species induced by transpiration and by exchange with cell wall binding sites has been found to promote light isotopes migration along the transport path [12,71].

Because the stem accounts for between ~80 and ~92% (depending on the habitat) of the above-ground larch biomass, the isotopic signature of the bulk wood approximates the isotopic composition of the whole larch above-ground biomass. Our results (section 4.3) demonstrate that the larch bulk wood collected from the South-facing slope is ~ 0.2‰ heavier than the nutritive topsoil and the bulk wood of the larch from a sphagnum peat bog is 0.2‰ lighter than the Sphagnum peat bog organic substrate of the rooting zone in this habitat. Therefore, there is a slight but measurable fractionation between the larch tree and the nutrient reservoir (mineral or peat soil). Various studies have shown that the whole vegetation could be enriched in light or heavy isotopes relative to the soil substrate depending on the element (e.g., Ca, Mg and Zn), the physiological status of the plant and the environment [20,33,72-78]. The trends observed for the larch in the South-facing slope in this study are consistent with those reported for tropical watershed [22], namely the plant enrichment in heavier Zn isotopes by 0.2-0.4‰ relative to the soil reservoir.

Interestingly, the single river water sample exhibited an isotopic composition similar to that of the larch needles, upper organic layers of the peat bog and the upper mineral soil horizons (0.2 ± 0.05‰). The isotopic measurements thus did not allow for discrimination between mineral and organic sources of Zn in surface waters within a 0.1‰ resolution. Curiously, this result corroborates recent conclusions achieved for Ca^47^, Si^45^ and Mg^48^ in this region; the stable isotope approach does not allow for a straightforward quantification of the relative contribution of plant litter, soil organic and mineral horizons and the bedrocks to the riverine fluxes of elements.

### Evolution of δ^66^Zn in the course of vegetative season

A recent study of nutrients and non-essential elements in larch needles during the vegetative season of the same boreal forest plot in Central Siberia [42] revealed that Zn micronutrient parallels the behavior of such macronutrients as nitrogen (N), phosphorus (P) or potassium (K) with decreasing concentration from June to September (see Figure 5). High concentrations of all these elements at the beginning of the growing period (June) are indicators of intense metabolic processes such as photosynthetic activity and protein synthesis when needles are first produced. The observed decrease of macro and micronutrient concentrations such as Zn between June and July can be explained by a dilution caused by C rather than Zn accumulation in the leafs whereas the decrease of Zn concentration between August and September results from a resorption [42]. The latter resorption process is a key mechanism for deciduous plants to avoid essential nutrient losses due to the litterfall. In fact, these elements can be mobilized towards the phloem in September at the end of the active period and before senescence and redistributed the next year to the younger tissues by the xylem.

Note that the Zn content in June should originate from the pool of metal accumulated at the end of the previous seasons as soil is still frozen in June. In the end of summer, the bulk isotopic signature can be considered as a mixture between a Zn pool stored in shoots from the previous year and a pool of Zn taken from the progressively thawing soils.

**Figure 7:**
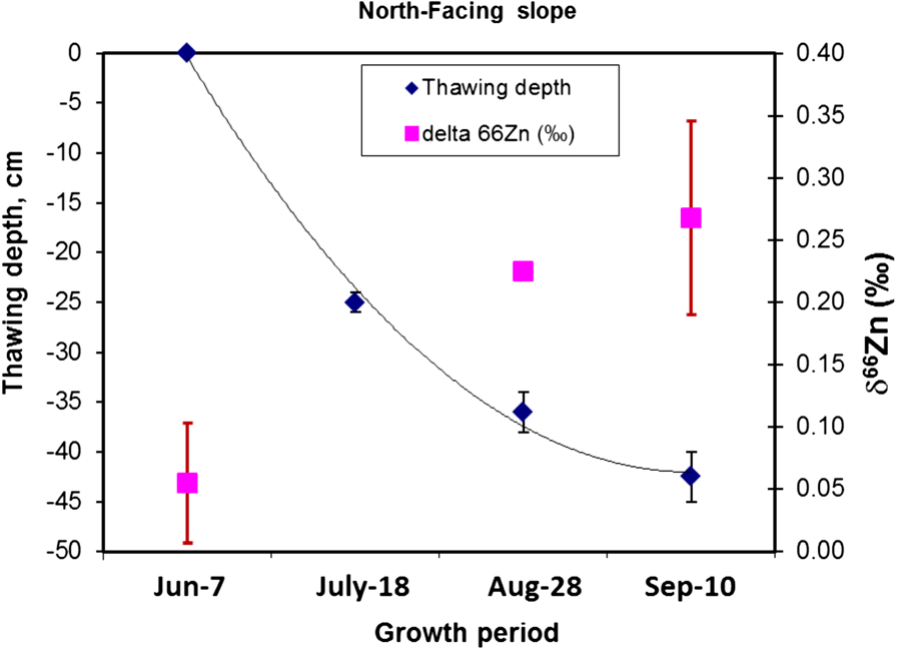
Evolution of the thawing depth and isotopic composition of larch needles as a function of time during the 2006 growing period (in case of the North-facing slope).

The zinc isotopic composition in larch needles as measured in early June is lighter than that of August or September and compared to that of the bulk wood (Figure 5 and Table 3). An enrichment of larch needles by a heavy Zn isotope at the end of the vegetative season relative to the beginning of the leaf growth may result from *1)* a change in the Zn source following the increase of the thawing depths, and/or *2)* fractionation induced by the metal transfer mechanism such as mobilization from the phloem to the leafs in early June and re-translocation at the end of the season. The first explanation is consistent with the fact that the soil thaws progressively from June to September (Figure 7). During this period, first, soil organic layer and then, deeper and deeper mineral soil horizons become involved in delivering Zn to the roots [79,80]. On the North-facing slope, there is an increase of δ^66^Zn from the surface (mosses) to the deeper layers (litter, organic layer, see Table 2). In the course of progressive thaw, a pool of heavier Zn will be delivered to the roots. The reason for this is that the soil solution in contact with a reservoir having δ^66^Zn = 0–0.1‰ at the beginning of the season will be isotopically lighter than the solution in contact with soil layers having δ^66^Zn = 0.2-0.4‰ later in the active season. The isotopic composition in the peat bog, where the rooting zone is always within the organic layer and not in the mineral horizon (see Table 1), also follows this scenario. In this setting, the heavier isotopes enrich the bottom soil horizons, which become progressively more available to the roots over the course of the season. As a result, the δ^66^Zn value of larch needles increases from August to September**.** The lighter isotopic composition of Zn in the needles and the bulk wood of the peat bog zone compared to slope environments could be explained by the higher dissolved organic carbon concentration in the solution in contact with this reservoir as it is known from previous works [44]. This higher DOC concentration in the interstitial solutions could make heavy isotopes less available for the larch roots compared to the settings of low DOC concentration, because the lower DOC will facilitate the uptake of heavy isotopes as it will be less retained in strong organic complexes.

A factor of 2 higher Zn concentrations in solution of SF soil compared to that of the NF slope (Figure 4) suggests more intense cycling of Zn in the warmer habitats. In deeper layers (BC/C, up to 60 cm depth which is close to permafrost) Zn concentrations in soil solution decrease to ca. 4 μg/L at both slope soils. Considering that all DOC of soil solution corresponds to fulvic acid we calculated the isotopic composition of the various Zn species when the DOC concentration increases from 10 to 50 mg/L with pH ranging from 3 to 6, encompassing the majority of soil solution composition. This calculation was performed for a constant Zn concentration of 10 μg/L considering that at the equilibrium the organic matter - complexed Zn will be 0.2‰ heavier than the free Zn^2+^ (see Jouvin *et al*. [65]). This first-order calculation shows that in acidic environment, the isotopic composition of the free Zn available for plants will be up to 0.1‰ lighter for a DOC concentration of 50 mg/L compared to 10 mg/L. This result is consistent with the measured difference of isotopic composition between the needles of the North-facing slope and the bog area.

The possible role of resorption processes on Zn isotope fractionation between June and September can be assessed from temporal evolution of Zn concentration in larch needles from various habitats. Zn concentration in the needles remained constant at the South-facing slope and bog and decreased between August and September at the North-facing slope. The carbon concentration in the needles decreased during the same period at the South-facing slope and bog or remains constant at the North-facing slope (Table 1) [42]. It follows that Zn resorption process involved between 10 to 25% of total Zn stock in the plant [42]. These values imply that for the resorption processes to influence the isotopic signatures at the level of 0.1 to 0.2‰, the δ^66^Zn isotopic shift corresponding to 10 to 25% of resorption should be on the order of 1.0 to 1.5‰, which is half a permil higher than the maximum Zn isotopic shifts reported during single-reaction adsorption or complexation processes [64,70]. As such, Rayleigh distillation processes should be invoked to explain isotopic fractionation by Zn resorption; however this could not be tested in this study.

Therefore, the most likely mechanism of Zn isotopic composition difference in the larch needles at the beginning and at the end of the vegetative season is the change of rooting depth in soil horizons upon soil thaw during active season, with rather uniform isotopic signature among contrasting habitats**.** At the same time, the obtained data did not allow to confirm the possibility that Zn is a limiting micronutrient for the larch. On slopes of contrasting nutritional status, the re-translocation affects Zn isotopic composition in needles in a variable degree, often comparable with the resolution of isotopic measurements.

Implying the previously developed approach of substituting space for time and assuming that the contemporary difference in local scales within the climate gradient created by slope orientation can serve as a proxy for future changes of a given system,^42,45,82^ results of the present study do not suggest any significant change in Zn isotopic pool of terrestrial biome in the Central Siberia at the most extreme scenario of climate warming in this region, i.e., in the case of complete replacement of N-facing cold and wet slope conditions by S-facing warm and dry habitats.

## Conclusions

This study revealed that Zn isotopes are not significantly fractionated within the permafrost-dominated soils of this site in Central Siberia. In contrast, measurable isotope fractionations were observed between the above-ground larch biomass and the soil reservoir. The isotopic composition (δ^66^Zn) of the larch needles depends on the habitat (North and South-facing slope or bog area) with 0.2-0.4‰ heavier values in the North- and South-facing slope relative to the peat bog. This trend is not related to a different physiological response of the tree but rather to the isotopic composition of the solution available to the rooting zone. It was hypothesized that stronger Zn complexation by organic ligands and humification products in bog areas leaves a soil solution which is isotopically lighter than the bulk mineral horizon.

Zn isotopes help to trace which free Zn pool is mobilized by the tree via following the rooting depth and soil solution chemistry in contrasted local environments and how it changes during the soil thawing over growing season. Specifically, during the course of the vegetation we observed enrichment in Zn heavy isotopes of larch needles. We suggest that the increased rooting depth and the decreased DOC concentration in the root uptake zone resulting from progressively thawing soil lead to heavy isotopes becoming more and more available for the larch roots in the course of the vegetative season. This is consistent with physico-chemical calculations showing that the decrease of DOC will facilitate the uptake of heavy isotope as it will be less retained in strong organic complexes. With the extreme climate evolution in Central Siberia, the North-facing slopes may experience climatic variations that are typical of present-day-South-facing slopes but it is unlikely that any significant change of Zn isotopic composition occurs in the soil-tree-river system.
